# Secular Trends in Prevalence and Risk Factors of Obesity in Infants and Preschool Children in 9 Chinese Cities, 1986–2006

**DOI:** 10.1371/journal.pone.0046942

**Published:** 2012-10-08

**Authors:** Xin-Nan Zong, Hui Li

**Affiliations:** Department of Growth and Development, Capital Institute of Pediatrics, Beijing, China; UCL Institute of Child Health, University College London, United Kingdom

## Abstract

**Introduction:**

No prevalence/trends were reported in Chinese infants and preschool children at the national level in this historical period of 1980s–2000s. The objectives of this paper were to present the 20-year trends in prevalence and risk factors of obesity in children below 7 years.

**Methods:**

Data obtained from a series of three sequential national surveys performed using the same design in the same 9 cities in 1986, 1996 and 2006. Weight and height (length <3 years) were measured using unified procedures at each period. Obesity was defined as a weight-for-height ≥120% of median of the NCHS/WHO international reference. A population-based paired matching case-control study was employed for screening risk factors.

**Results:**

In 1986, there was no substantial obesity epidemic, but the overall obesity prevalence reached 3.4% (boys 4.1% and girls 2.7%) in 2006, rising by 2.8 times between 1986 and 2006. Reversed gender difference, relatively higher prevalence in preschool age and more rapid increase in the second 10-year were three very obvious characteristics in China, e.g. prevalence of boys 9.9% and girls 4.9% in 2006 and increasing rate of boys 0.45 percentage points per year (pp/y) and girls 0.21 pp/y at 6–7 years groups, 0.17 pp/y of the second 10-year higher 1-fold than previous.

**Conclusions:**

China has been moving into the alarming epidemic of childhood obesity. Effort should be immediately made to prevent further deterioration. High birth weight, high parental BMI and several behavioral and family-related factors were identified and had important practical value for obesity intervention.

## Introduction

Childhood obesity increased tremendously worldwide during the last decades [1]–[3]. In children and young people, the short-term deleterious effects of obesity on physiological responses have been well documented as an unfavorable impact on blood pressure, blood lipids and blood glucose [4]–[6]. Moreover, more disappointing is that obese children are at high risk of becoming obese adults [7], their associated with chronic conditions, such as diabetes or hypertension or coronary heart disease [8]–[9]. Obese children are also susceptible to some psychosocial and behavioral complications [10]–[11]. So many countries/regions/organizations attach great importance to estimation of prevalence and analysis of risk factors of obesity in childhood for helping health policy planners develop intervention strategies to overcome strong influences of obese environments at young ages.

**Figure 1:**
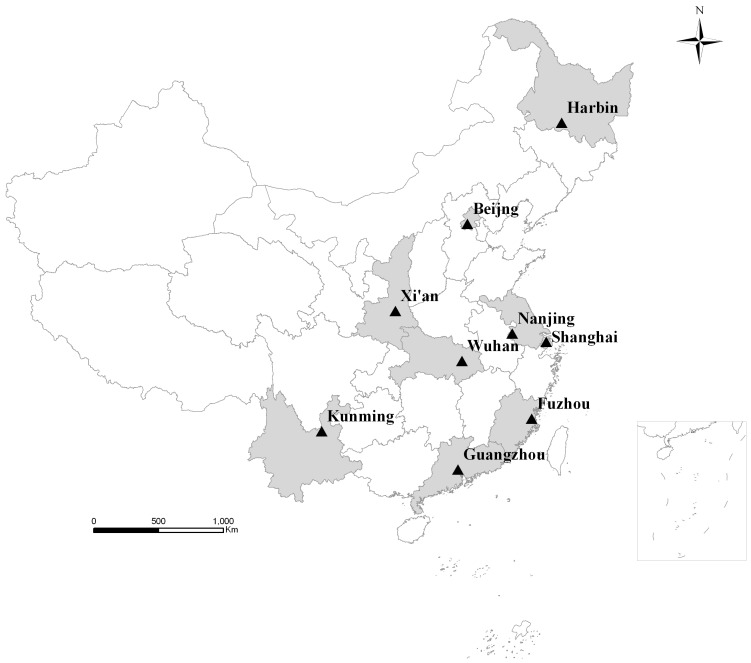
Geographical distribution of the 9 cities (Shaded their corresponding provinces) in China.

In China, the National Epidemiological Survey on Simple Obesity in Childhood (NESSOC) was carried out for the first time in 8 major cities in 1986 [12], including Beijing, Harbin, Xi’an, Shanghai, Nanjing, Wuhan, Fuzhou and Kunming (Figure 1). This survey involved about 140 thousand children below 7 years of age and obtained the 1^st^ national epidemiological baseline data of prevalence and risk factors of simple obesity in childhood. Thereafter, to further study the epidemic secular trends, the 2^nd^ and 3^rd^ NESSOC were conducted using the same study design and the same reference defining obesity in the aforementioned cities in 1996 [13] and 2006 [14] respectively, and the city Guangzhou also participated in these two surveys.

Epidemic increase in overweight and obesity was reported for Chinese children aged 7–18 years from 1985 to 2005 [15], but almost no prevalence/trends for Chinese infants and preschool children at the national level in this historical period. Trends in obesity could not be systematically assessed from childhood to adolescence in China because of the shortage of this indispensable data. Therefore, the aim of this paper was (a) to describe epidemiological characteristics and secular trends of prevalence of obesity and (b) to report unchanged and changed risk factors of obesity in infants and preschool children on the basis of three sequential national surveys in China between 1986 and 2006.

## Subjects and Methods

### Survey Locations, Sampling and Subjects

The NESSOC was carried out in 9 major cities (Figure 1) in China in 1986, 1996 and 2006 respectively. Of these, Beijing and Shanghai are municipalities, and the other seven are provincial capital cities, Harbin (Heilongjiang’s provincial capital), Xi’an (Shaanxi), Nanjing (Jiangsu), Wuhan (Hubei), Guangzhou (Guangdong), Fuzhou (Fujian), and Kunming (Yunnan).

Multistage stratified cluster sampling was used in this survey. One or more districts in these cities were selected as the study units, and the estimated numbers of children below 7 years were more than 10 thousand. All children of Han ethnicity below 7 years, living in these study units represented the study subjects. Children below 3 years were selected from street community as minimum cluster unit and those over 3 years (including 3 years) from kindergarten as cluster unit. Because children at age 3 years or older attended kindergartens and started primary school at 7 years. The number of communities and kindergartens were estimated based on the total number of children below 7 years and its age distribution in the selected districts of each city. The participation rate was not less than 95% in the selected communities and kindergartens, and the participants were not less than 10 thousand in each city [16].

### Definition and Screening of Obesity

Overweight was defined as a weight-for-height ≥110% of median of NCHS/WHO reference [17], obesity as ≥120% for mild obese, ≥130% moderate, ≥150% severe. In the 1970s China constructed its own child health care system. The network contains thousands of medical staff and hundreds of child healthcare centers or institutes. Weight and height values of each child from birth to 7 years are measured regularly (annually or semiannually) through this network. These values are documented in the child’s health record and taken as a crude screening basis in our studies. Current weight and height measurements were taken in the field sites of kindergartens and child health care center for final identification and classification of degrees. Any extreme values caused by measurement errors can be easily identified and corrected immediately according to the child’s health record. Secondary or pathological obesity was identified by senior doctors according to “inclusion and exclusion criteria of childhood simple obesity” for separate statistics, if necessary, assistant or clinical examinations were added.

### Control Group and Questionnaires

A population-based case-control study was employed for screening risk factors of childhood obesity. Due to the variation of potential risk factors among different ages and gender, one identified obese child was matched with one randomly selected non-obese child from the same child health center, by using the individual child’s health record for the same gender, similar age (difference less than 6 months when children over 1 years, less than 2 months when 6 months to 1 years, less than 1 month when under 6 months), and similar height (difference within ±3 cm). Potential risk factors were chosen based on previously reported associations, or plausible prior hypotheses, including infant status at birth, maternal condition during pregnancy, feeding pattern and eating habits, child activity, behavior and habits, and family background, etc [16].

### Data Collection and Quality Control

Data collection was done separately by the study subgroups in the 9 cities. The number of children investigated was listed by gender and age groups (1mon-, 1 yr-, 2 yrs-, 3 yrs-, 4 yrs-, 5 yrs-, 6–7 yrs) according to the rosters of communities and kindergartens through the network. Weight and height of all children were measured in a standardized way by specially trained technicians or nurses [18]. The height of each child was measured to the nearest 0.1 cm as supine length before 3 years of age and as standing height (not in shoes) after that. The weight was obtained to the nearest 0.01 kg with children wearing the lightest vest, shorts or underwear. Weight error was not more than 0.05 kg and height not more than 0.5 cm among groups and between two repeated measurements. For children who attended kindergarten, their parents were met in the kindergarten when they picked up their child. Part of the information about the daily activity of the child was reported by kindergarten teachers. For other children from communities, parents were asked to take their children to a certain local child health care center to meet our study group. Questionnaires concerning the activity and feeding pattern of the child, and other parental information such as education level, attitude to obesity and their current weight and height were administered to the parents on the day of investigation. More details about questionnaires could be obtained from the book [17]. All completed study forms and questionnaires were required to carefully check and to send to the coordinating study group of Beijing for statistical analysis, including forms for actual number of investigation and for simple overweight, forms and questionnaires for simple obesity and card for secondary obesity. The electronic database was established for forms, cards and questionnaires by double entry.

### Organization and Time of Survey

The Capital Institute of Pediatrics, the oldest and most important pediatric research institutes in China was appointed to organize and coordinate the series of NESSOC by the Ministry of Health since 1986. Under the direction of the Beijing Steering Committee, the 9 coordinating study subgroups worked simultaneously, following a uniform plan, organizing and training local survey teams, carrying out the measurements and questionnaires. Field investigators must participate in rigorous training and pass the examination before starting the survey who were physicians/pediatricians, nurses and technicians. Unified measuring tools/instruments were equipped to minimize the influence of measurement errors for each field site. The survey started in June and finished in October of the same year. All the measurements were undertaken at least one hour after a meal, between approximately 8 a.m. and 4 p.m.

### Statistical Analysis

The increasing rates in obesity were expressed using two methods, proportional increase, percentage (%) and absolute increase, percentage points per year (pp/y). Differences in obesity prevalence were tested for significance using Chi-square tests between genders and among three surveys. Conditional logistic regression was used to examine potential risk factors by stepwise selection and 0.05 for sizes of a test, and an odds ratio (OR) significantly (P<0.05) higher than 1.00 was regarded as a risk factor of obesity. Data was analyzed with SPSS, version 13.0 (SPSS, Inc., Chicago, Illinois).

## Results

To improve comparability of data, we mainly described distributions and trends in obesity in 8 cities (not including Guangzhou) from 1986 to 2006. The number of sample size was 138,029 in 1986, 109,701 in 1996 and 96,398 in 2006, and the ratio of male to female ranged from 1.04 to 1.16 in all age groups, more details about age distribution in Table 1.

**Table 1:** Trends in obesity prevalence in age, 1986–2006.

Age	N a			Prevalence (%)			Proportional increase (%)			Absolute increase (pp/y b)		
	1986	1996	2006	1986	1996	2006	1986–1996	1996–2006	1986–2006	1986–1996	1996–2006	1986–2006
1mon-	27605	12642	13151	1.65	1.23	1.90 c	−25.5	54.5	15.2	−0.04	0.07	0.01
1 yr-	19427	14238	13723	0.55	0.54	1.00 c	−1.82	85.2	81.8	0.00	0.05	0.02
2 yrs-	18908	14523	12323	0.36	0.46	1.18 c	27.8	156.5	227.8	0.01	0.07	0.04
3 yrs-	21078	16037	13395	0.35	0.75	2.02 c	114.3	169.3	477.1	0.04	0.13	0.08
4 yrs-	19132	17300	16306	0.49	1.49	3.91 c	204.1	162.4	698.0	0.10	0.24	0.17
5 yrs-	15669	18423	16717	0.84	3.07	6.05 c	265.5	97.1	620.2	0.22	0.30	0.26
6–7 yrs	16210	16538	10783	0.84	4.17	7.64 c	396.4	83.2	809.5	0.33	0.35	0.34
Average	138029	109701	96398	0.91	1.76	3.44 c	93.4	95.5	278.0	0.09	0.17	0.13

Results shown are representative of the 8 cities surveyed, not including Guangzhou.

a N, numbers of subjects.

b pp/y, percentage points/year.

c Chi-square test for trend, P<0.01.

### General Trends

The obesity prevalence increased rapidly from 0.91% to 3.44%, rising by 2.8 times during the 20 years. In the second 10-year, the increasing rates in obesity of 95.5% and 0.17 pp/y were higher than those of 93.4% and 0.09 pp/y in the first (Table 1).

### Trends in Gender and Age

**Figure 2:**
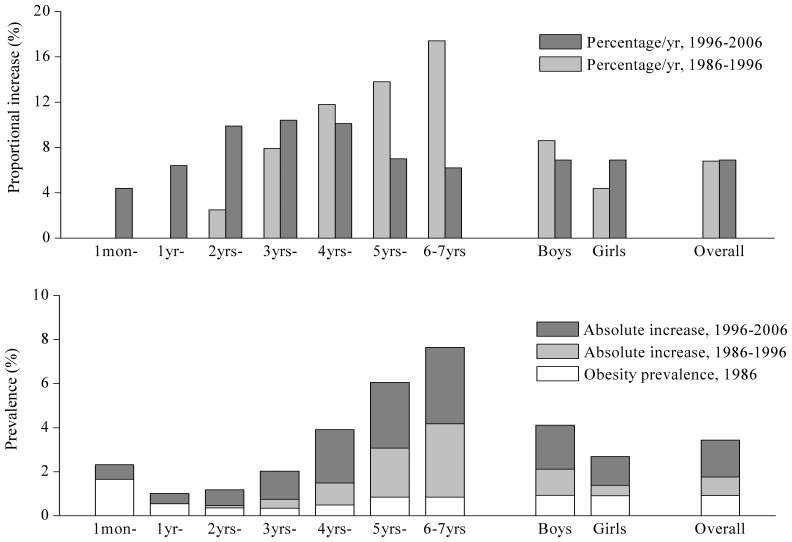
Trends in obesity prevalence based on the 8 cities in China, by gender and age, 1986–2006.

There was no significant gender difference in obesity prevalence between boys 0.93% and girls 0.90% in 1986, but boys’ higher than girls’ in 1996 and 2006. The increasing rate in obesity was boys 343.0% (0.16 pp/y) and girls 198.9% (0.09 pp/y) during the 2 decades. The obesity prevalence was relative lower in almost all age groups in 1986, but rose sharply in the next 20 years, especially at over 4 years of age, for example, rising by 0.45 pp/y for boys and 0.21 pp/y for girls at group 6–7 years. Furthermore, the increasing rate in the second 10-year was strikingly faster than that in the first in all age groups, similar for boys and girls (Tables 1 and 2, Figure 2).

**Table 2:** Trends in obesity prevalence in gender and age, 1986–2006.

	Prevalence (%)						Absolute increase (pp/y a)					
Age	Boys			Girls			Boys			Girls		
	1986	1996	2006	1986	1996	2006	1986–1996	1996–2006	1986–2006	1986–1996	1996–2006	1986–2006
1mon-	1.85	1.49	2.18 b	1.43	0.97	1.62 b	−0.04	0.07	0.02	−0.05	0.07	0.01
1 yr-	0.49	0.62	1.17 b	0.62	0.46	0.81 b	0.01	0.06	0.03	−0.02	0.04	0.01
2 yrs-	0.40	0.49	1.17 b	0.32	0.43	1.18 b	0.01	0.07	0.04	0.01	0.08	0.04
3 yrs-	0.28	0.72	2.07 b	0.43	0.78	1.96 b	0.04	0.14	0.09	0.04	0.12	0.08
4 yrs-	0.48	1.71	4.37 b	0.51	1.25	3.43 b	0.12	0.27	0.19	0.07	0.22	0.15
5 yrs-	0.84	3.58	7.40 b	0.83	2.51	4.54 b	0.27	0.38	0.33	0.17	0.20	0.19
6–7 yrs	0.89	5.31	9.86 b	0.79	2.80	4.94 b	0.44	0.46	0.45	0.20	0.21	0.21
Average	0.93	2.12	4.12 b	0.90	1.38	2.69 b	0.12	0.20	0.16	0.05	0.13	0.09

Results shown are representative of the 8 cities surveyed, not including Guangzhou.

a pp/y, percentage points/year.

b Chi-square test for trend, P<0.01.

### Trends in Cities

**Figure 3:**
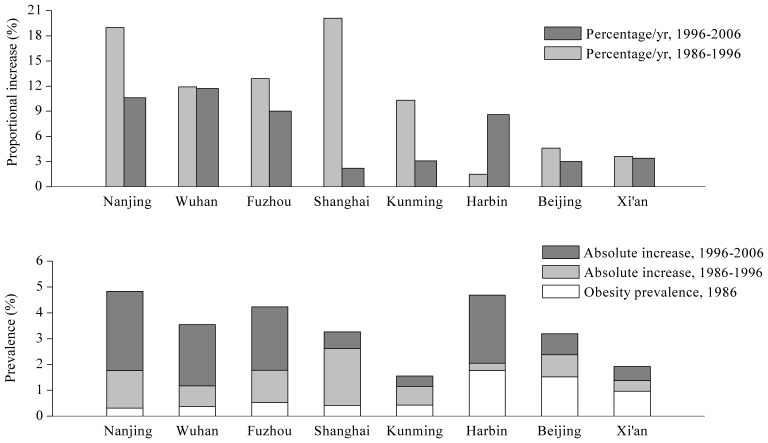
Trends in obesity prevalence based on the 8 cities in China, by city, 1986–2006.

The top three cities in obesity prevalence were Nanjing 4.83%, Harbin 4.69% and Fuzhou 4.23% in 2006. In the first 10-year, the top three cities in increasing rates of obesity were Shanghai 523.8% (0.22 pp/y), Nanjing 467.7% (0.15 pp/y) and Fuzhou 235.8% (0.13 pp/y); but in the second, the higher increasing rates were seen in Nanjing 0.31 pp/y, Harbin 0.26 pp/y, Fuzhou 0.25 pp/y and Wuhan 0.19 pp/y. Whereas the lowest increasing rates were Xi’an 0.05 pp/y and Kunming 0.06 pp/y on average during the two 10- year (Table 3, Figure 3).

**Table 3:** Trends in obesity prevalence in cities, 1986–2006.

City	Prevalence (%)			Proportional increase (%)			Absolute increase (pp/y a)		
	1986	1996	2006	1986–1996	1996–2006	1986–2006	1986–1996	1996–2006	1986–2006
Beijing	1.52	2.38	3.19 b	56.6	34.0	109.9	0.09	0.08	0.08
Harbin	1.76	2.05	4.69 b	16.5	128.8	166.5	0.03	0.26	0.15
Xi’an	0.97	1.38	1.92 b	42.3	39.1	97.9	0.04	0.05	0.05
Shanghai	0.42	2.62	3.26 b	523.8	24.4	676.2	0.22	0.06	0.14
Nanjing	0.31	1.76	4.83 b	467.7	174.4	1458.1	0.15	0.31	0.23
Wuhan	0.38	1.17	3.03 b	207.9	159.0	697.4	0.08	0.19	0.13
Fuzhou	0.53	1.78	4.23 b	235.8	137.6	698.1	0.13	0.25	0.19
Kunming	0.43	1.15	1.56 b	167.4	35.7	262.8	0.07	0.04	0.06

Results shown are representative of the 8 cities surveyed, not including Guangzhou.

a pp/y, percentage points/year.

b Chi-square test for trend, P<0.01.

### Trends in Degree of Obesity

Mild and moderate were in the majority. Mild obesity rose from 0.50% in 1986 to 1.86% in 2006, by 2.7 times, moderate from 0.30% to 1.22%, by 3.1 times. Severe obesity rose from 0.10% to 0.22%, and most of which mainly occurred in over 4 years old.

### Trends in Risk Factors, 1996–2006

To maximize use of existing data, data from 9 cities of the 1996 and 2006 surveys was used to analyze potential risk factors of obesity. Table 4 showed that birth weight, maternal BMI, paternal BMI, current appetite, eating speed, intensity of outdoor activities were closely related to obesity in the 1996 and 2006 surveys. Night sleep duration, time spending watching TV, types of delivery and staffs of child caretaker also entered multivariate regression model in the 2006, but feeding patterns in the first 4 months in 1996 was not screened out in this survey.

**Table 4:** Associations between risk factors and obesity in childhood using multivariate conditional logistic regression model, 1996–2006.

Survey 1996		Survey 2006	
Risk factors	OR (95%CI) b	Risk factors	OR (95%CI)
Birth weight, continuous (100 g units)	1.051(1.021–1.082)	Birth weight, continuous (100 g units)	1.016(1.002–1.031)
Feeding patterns in the first 4 months,binary (breastfeeding, milk/milkpowder as control ) c	0.636(0.452–0.896)	Types of delivery, binary (caesarean, vaginalas control )	1.508(1.222–1.863)
Father’s BMI, continuous (1 kg/m^2^ units) d	1.211(1.144–1.281)	Father’s BMI, continuous (1 kg/m^2^ units)	1.080(1.043–1.118)
Mother’s BMI, continuous (1 kg/m^2^ units) d	1.226(1.159–1.297)	Mother’s BMI, continuous (1 kg/m^2^ units)	1.107(1.066–1.148)
Current appetite, ordinal (good,average, bad)	6.413(4.760–8.640)	Current appetite, ordinal (good, average,bad)	4.936(3.854–6.320)
Fast eating speed, dummy (averageas control)	6.389(4.139–9.864)	Fast eating speed, dummy (average ascontrol)	3.986(3.068–5.179)
Types of activities: outdoor’s,categorical (outdoor’s, indoor’s,watching TV as control)	0.700(0.505–0.971)		
Types/intensity of outdoor activities:running, categorical (running,walking, sitting as control) e	0.701(0.516–0.953)	Types/intensity of outdoor activities:running, categorical (running, walking,sitting as control)	0.674(0.539–0.842)
		Time spending watching TV, ordinal(<0.5 hr, 0.5–1 hr, 1–2 hrs, >2 hrs)	1.187(1.060–1.329)
		Duration of night sleep time,continuous (0.5 hr unit)	0.925(0.861–0.994)
		Staffs of child caretaker: parents,categorical (parents, grandfathers orgrandmothers, nannies as control)	0.667(0.526–0.845)

Data aged 2–7 years from the 9 cities was used for analysis, with 1016 pairs in 1996 and 1593 pairs in 2006, because of the full coverage of factors surveyed in this age range.

Conditional logistic regression was used to screen potential risk factors by stepwise selection and 0.05 for sizes of a test.

a *β* represented parameter estimate with positive value as possible risk factors and negative value as possible protective factors.

b OR, Odds ratio; CI, Confidence interval.

c Breastfeeding includes exclusive and partial with 〉1/2 of total intake.

d Body mass index (BMI) is calculated as weight (kg) divided by height (m) squared through self-reported weight and height.

e Running includes running, jumping, climbing stair, et al; sitting represented not or hardly playing when others doing.

## Discussion

From a global perspective, childhood obesity has long been associated with western countries and authorities view it as one of the most serious public health problems of the 21^st^ century [19]. However, the rapid increasing trend had spread to developing countries in two more decades. In developed societies, the obesity prevalence increased by about 2 to 4 times from 1970s to 1990s [20]–[22], and recently at a much higher level [23]–[25]. On the other hand, the obesity prevalence was as high as 3.3% in developing countries in the late 1990s [26], and over 40% of these indicated the rapid increase with trend data since 1990 [27]. As a big developing country, China experienced a rapid nutrition transition and dietary change [28]–[29] after the establishment of market economy system in the early 1990s. The rising population obesity might therefore be expected [15], [30] which should be attributed to increases in energy and fat intake and decreases in physical activity.

In 1980s, there was no substantial obesity epidemic in China. Unfortunately, the increasing trend over time was alarming in our series of studies, with an increase of 5–8 times for 3 yrs- to 6–7 yrs groups in the two decades. In particular, the higher increasing rate was seen in all age groups in the second 10-year, e.g. higher 0.1 pp/y than that in the first for 3 yrs- to 5 yrs- groups. In 2006, the overall prevalence of obesity was 3.4% in infants and preschool children, and 6.1% for 5 yrs- groups and 7.6% for 6–7 yrs groups reached an alarming level. The increasing rates of boys’ 0.45 pp/y and girls’ 0.21 pp/y showed a shocking epidemic during the 20 years. In fact, China has reluctantly entered the obesity epidemic stage. More practical and effective measures should be immediately taken to prevent further deterioration.

From an epidemiological perspective, childhood obesity varied by gender, age and city. We found some interesting characteristics of prevalence and secular trends of obesity in infants and preschool children in China. The gender indifference was a very obvious characteristic for Chinese populations and boys’ prevalence/increasing rate of obesity were appreciably higher than girls’, which were also verified by other surveys among teenagers in China [15], but different from western studies [20], [22], [31], even from Japan also located in East Asia [32]. This characteristic was also verified by comparison of weight and body mass index (BMI) between both genders and among the China references, the WHO curves and the U.S. CDC2000 curves [33]–[34]. Disparity of gender difference in obesity between China and western countries was an interesting fact and has given rise to our concern, perhaps suggesting diversification of race/ethnicity, geography/climate and traditional cultural values.

Another notable characteristic was high prevalence/increasing rate of obesity mainly occurred in preschool children but not infants. This increasing trend with age was also verified by a three-year follow-up study on preschool obesity [35]. Generally, a relative low level stayed right there in infants in the first 10-year, but the growing trend appeared in the second 10-year, e.g. 156.5% at 2 yrs- group. In China, breastfeeding was the primary feeding pattern, the proportion of exclusive (overall) breastfeeding was 53.5% (86.2%) for infants below 6 months of age in the 9 cities in 1995, but reduced to 32.8% (79.9%) in 2005 [36]. The obesity prevalence and its change in infancy may be related to breastfeeding and its change. On the other hand, more attention should pay to a high prevalence/increasing rate in preschool children. We think that this performance may be linked to the two changes. Firstly, in the ages children began to have lifestyle of resting, more time spending TV, video games and computers and participating in training courses, such as Olympic Mathematics, foreign languages and painting. Secondly, children in the ages began to get more Western and fast food. The imbalance in energy intake and consumption by the two changes may be an important cause of obesity. However, it is not very clear for this association and perhaps additional study is required to further identify.

When it comes to regional differences in obesity, our concern is not simply high or low. Because clear geographical variation may be mainly caused by disparity of socioeconomic status and its related dietary and lifestyle changes in such a geographically vast country [37]. In all cities surveyed, the growing obesity epidemic was observed in the two decades. In particular, in most instances, the increasing rate was much faster in the second 10-year than that in the first, such as Nanjing, Harbin, Fuzhou and Wuhan. Perhaps it suggested that prevention strategies for childhood obesity to date had usually been unsuccessful and were necessarily modified. Encouragingly, the increasing rate was somewhat lower in the second 10-year in Shanghai, and Shanghai local monitoring also confirmed the existence of such a decreasing change, suggesting effective interventions could control morbidity of obesity [38].

From a developmental perspective, obesity has its origins in early life. There may be vulnerable periods for weight gain during childhood. Once children are obese, it is often difficult for them to lose weight through physical activity and healthy diet. It is therefore recognized as a feasible strategy that controlling rapid weight gain depending on regular growth monitoring and permanent changes in lifestyles from an early age or even from pregnancy and infancy onward [27]. The identified risk factors by our repeatable studies in different periods were very important practical value for intervention program of obesity in childhood. High birth weight, mother’s high BMI and father's high BMI were identified as risk factors which were also confirmed by other cohort studies [39]–[40]; Lifestyle and physical activity, such as current appetite, eating speed and types/intensity of outdoor activities were also closely related to obesity in preschool children. The new findings through the 2006 survey were night sleep duration, time spending watching TV and staffs of child caretaker. In addition, we should also be aware of other two changes. Breast-feeding was not significant between obesity and control groups with OR = 0.825 (95%CI, 0.635–1.071) by comparing with previous studies in the 1996, we will continue to concern this disappeared change. The univariate analysis showed the differences of parental education between control and obesity groups were statistically significant (P<0.01) in the 1996 and 2006 surveys. However, it did not enter multivariate logistic model at 0.05 level.

An interesting phrase emerged: family-related factors when we reorganized these identified risk factors. Broadly speaking, it included not only family-related environment, lifestyle and behavioral characteristics, but also family history of obesity. This concept has a strong operability for identifying high-risk groups and taking family-based intervention in preschool age. We hope it helps to promote healthy development of children and control of obesity.

To facilitate comparison to other studies, here we give some prevalence of obesity using more common definitions from Cole et al [41]. The crude obesity rates were 1.28% (boys 1.63% and girls 0.90%) in 1996 and 2.28% (boys 2.95% and girls 1.54%) in 2006 for children 2–6.9 years of age in the 9 cities. The increasing rate was 78.4% and 0.10 pp/y in the 10-year. Obviously, the prevalence of obesity using the Cole’s cut offs was lower than that using the previous NCHS/WHO reference.

### Limitations of this Study

There were three limitations arising from the historical conditions. Guangzhou did not participate in the 1^st^ survey in 1986, thus secular changes of obesity prevalence were described only using those data from 8 cities (Guangzhou excluded) to optimize the comparability between 1986 and 2006. No overweight prevalence/trends were presented because of the incongruous overweight screening, defined as a weight-for-height ≥115% of median of NCSH/WHO in 1986 and 1996, adjusted to 110% in 2006. Risk factors could not be analyzed using the multivariate model from 1986 as a result of shortage of original dataset and questionnaires.

### Implications

Rapid obesity increase was observed in infants and preschool children between 1986 and 2006 and China has been moving into the alarming epidemic of childhood obesity. Effort should be immediately made to prevent further deterioration. Epidemiological distribution and trends in obesity had Chinese characteristics. This concept of family-related factors has a strong operability for identifying high-risk groups and taking family-based intervention. In this paper, secular trends of prevalence and risk factors of obesity in childhood are of importance not only to develop multiple and integrated interventions to stem the rising tide of obesity in this country and but also to further explore distribution and estimation of obese children around the world.
